# Home-based cognitive bias modification training for reducing maladaptive fear in patients with suspected acute coronary syndrome: a pilot randomized clinical trial

**DOI:** 10.1186/s40814-024-01442-2

**Published:** 2024-01-11

**Authors:** David Lopez-Veneros, Robin Cumella, Ian M. Kronish, Amit Lazarov, Jeffrey L. Birk

**Affiliations:** 1 Department of Medicine, Center for Behavioral Cardiovascular Health, Columbia University Irving Medical Center, 622 West 168th Street, New York, NY 10032 USA; 2 School of Psychological Sciences, Tel Aviv University, P.O. Box 39040, 6997801 Tel Aviv, Israel; 3 Department of Psychiatry, Columbia University Irving Medical Center, 1051 Riverside Drive, New York, NY 10032 USA

**Keywords:** Feasibility, Cognitive bias modification training, Intervention, Acute coronary syndrome, Fear of recurrence

## Abstract

**Background:**

Patients evaluated in an emergency department for suspected acute coronary syndromes (ACS; e.g., myocardial infarction) often experience a lingering fear of recurrence, which may adversely affect their mental health and adherence to recommended health behaviors. Cognitive bias modification training (CBMT) is an acceptable, easy-to-use intervention that reduces fear of recurrence in cancer patients, and reduces fear and anxiety in other populations, providing an alternative to psychotherapy or counseling-based approaches. Feasibility testing is needed to assess whether a cardiac-related version of CBMT is acceptable to patients with elevated threat perceptions related to their suspected ACS.

**Methods:**

We developed a tablet-based CBMT intervention tailored to reduce cardiac-related fear of recurrence. In this double-blinded feasibility trial, patients with elevated threat perceptions related to a recent suspected ACS were randomized either to a 4-week, 8-session, tablet-delivered intervention (CBMT) group or to a sham attention control group. Feasibility outcomes included the proportion of eligible patients who enrolled, drop-out rate, intervention compliance rate, acceptability/pleasantness and usability ratings, and task engagement (i.e., accuracy, response time).

**Results:**

Of 49 eligible patients with suspected ACS and elevated threat perceptions recruited from NewYork-Presbyterian Hospital, over half (53.1%) enrolled after receiving a description of study procedures. Of the 26 randomized patients (mean age 59.15 years, 50% women), 2 patients (7.7%) dropped out. Additionally, 4 (15.4%) enrolled patients were not able to complete the tablet tasks, either due to difficulties with the technology or an inability to process the visually presented linguistic information at a sufficient speed. Still, among patients who returned the tablets (19 returned/20 received; 95%), most completed all assigned tablet tasks (intervention or control; 10/19; 52.6%), reporting that the tablets were easy to use and that the tasks were pleasant to complete.

**Conclusion:**

Current findings suggest that cardiac-related CBMT is a promising and generally acceptable intervention for suspected ACS patients with cardiac-related threat perceptions which are akin to fear of recurrence. Nevertheless, challenges related to tablet usage indicate that the intervention user-experience should be further refined to optimize usability.

**Trial registration:**

Registered at ClinicalTrials.gov on 2/25/2019; NCT03853213. Registered with the Open Science Framework on 11/20/2017; https://osf.io/k7g8c/.

## Key messages on feasibility

- What uncertainties about feasibility existed?
  It was unknown whether patients with elevated threat perceptions surrounding evaluation for suspected acute coronary syndrome would be willing to initiate and adhere to a cognitive bias modification training (CBMT) intervention involving cardiac-related stimuli, as these could serve as aversive reminders of a potentially traumatic medical event. It was also unknown whether randomization and blinding would be acceptable to patients. Finally, the feasibility of the return of intact electronic tablet devices was uncertain.
- What are the key feasibility findings?
  The CBMT intervention adapted for cardiac fear of recurrence delivered via electronic tablets showed moderate evidence of being acceptable to patients with a recent hospitalization for suspected acute coronary syndrome as only about half of eligible patients agreed to participate. The overall findings were mixed; however, regarding the feasibility of administering the intervention via tablets, a sizable group of participating patients were not able to complete the key study procedures. On the one hand, over two thirds of patients who finished the study completed most of the requested self-administered intervention sessions. On the other hand, a sizable proportion of patients (23.1%) were either unable to follow the study procedures, due mainly to technology discomfort (15.4%) or study attrition (7.7%). Still, completing patients rated the tablet-administered intervention as generally pleasant and very easy to use.
- What are the implications of the feasibility findings for the design of the main study?
  The intervention’s content, session duration, and number of assignments were generally acceptable to this patient population. Critically, however, the intervention’s technological and practical aspects were not sufficiently feasible to administer to this entire patient population. The authors recommend that researchers who pursue this intervention in a large clinical trial administer the sessions via a different modality that requires no patient interaction with an unfamiliar device and no need for device return (e.g., smartphone app, HTML-based software), incorporate stricter eligibility criteria, and/or include a run-in period prior to preliminary efficacy testing.

## Background

The majority of patients hospitalized for a suspected acute coronary syndrome (ACS; unstable angina or myocardial infarction) experience a fear of death in the emergency department [1] lingering for days after the event [2]. Unfortunately, some patients’ emotional distress is high and does not resolve over time. Approximately 10% of these patients suffer from clinically debilitating levels of distress in the weeks and months after hospitalization [2, 3]. This pattern occurs even among patients who are ultimately ruled out for ACS [1, 4]. Recent work reveals that many patients evaluated for ACS or other acute cardiovascular conditions greatly fear the progression of their illness and that this fear strongly predicts the development of posttraumatic stress symptoms [5], which in turn may be associated with an increased risk for a secondary cardiac events or even death [6].

The growing area of research on fear of recurrence (FoR) in cardiac populations should be informed by the large body of work on FoR in patients with cancer—over 50% of people who receive a cancer diagnosis develop a lasting FoR [7], which reduces patients’ quality of life [8, 9] and adversely influences their health behaviors, such as reductions in physical activity and increases in alcohol consumption [10]. The parallel literature on cardiac FoR, however, is less extensive. Cardiac-related FoR includes serious concerns about pain, damage to one’s body, disruption of professional goals, burden on family members, troublesome medical treatments, and death [5]. State anxiety within 2 weeks of an ACS event is robustly associated with lower quality of life 12 months following the event [11]. The intensity of fearful perceptions experienced during the acute event predicts the development of posttraumatic stress disorder (PTSD) a month later among patients with anxiety about their cardiac sensations [12]. In turn, cardiac-induced PTSD symptoms are associated with a doubled risk of having another acute coronary event or dying within the next 12 months [13].

FoR may be associated with such poor outcomes in part because it promotes poor health behaviors due to a maladaptive emotion regulation strategy of fearful avoidance. Indeed, fearful cardiac patients tend to avoid taking their prescribed medications because they serve as aversive reminders of their medical condition [14, 15], and they tend to avoid physical activity due to cardiac-related fear from internal bodily sensations, such as rapid heartbeat or shortness of breath[16], each of which may put them at a higher risk of having another acute event. Thus, there is reason to believe that cardiac FoR is not merely a highly unpleasant nuisance but may worsen long-term mental and physical health outcomes, highlighting the urgent need to screen for and develop targeted interventions to reduce FoR in ACS survivors [17]. Considerable work has been done to conceptualize FoR in cancer populations [18] and to target the construct via intervention in those cancer survivors, with varying degrees of success [19–22]. Initial work suggests that FoR may be similar in other medical populations as in cancer populations [23], but little work has yet tested FoR interventions in fearful cardiac patients. This intervention research may not only improve mental and physical health outcomes, but its use of an experimental method allows for a causal test of whether cardiac FoR might be a critical mechanism of health behavior change in this patient population [24, 25].

A promising intervention for reducing cardiac-related FoR is cognitive bias modification training (CBMT). This intervention was developed to reduce psychological distress by retraining two underlying cognitive factors that are robustly associated with and believed to contribute to dysfunctional emotional outcomes, including chronic fear and anxiety. The first cognitive factor is the habitual preference to attend to negative information [26, 27]. The second factor is the tendency to assign negative meaning to ambiguous stimuli [28, 29]. The full version of the CBMT intervention thus includes both the cognitive bias modification of attention (CBM-A) and of interpretation (CBM-I) [30]. CBMT has been delivered through a variety of platforms, including in-person lab training [31], web-based training [32], and out-of-lab training via mobile devices [33] and tablets [34]. CBMT has shown promise in the reduction of anxiety and depressive symptoms and has been investigated for many other conditions, including PTSD, panic disorder, generalized anxiety, obsessive-compulsive disorder, social anxiety, and anxiety sensitivity [30, 35–40], and various conditions in which cognitive tendencies are believed to underlie or worsen the disorder (e.g., chronic pain, paranoia, alcohol use disorder) [41–43]. It has shown varying levels of efficacy across studies. Regarding the target of anxiety, which is the closest available construct to FoR, the current estimate for CBMT’s anxiety reduction is significant with a small effect size (Hedges’ *g* = 0.23) [44]. Notably, a large recent network meta-analyses (85 trials; 3897 participants) showed that the CBM-I component, in particular—though not the CBM-A component—had reliable effects in reducing anxiety when tested relative to a sham treatment or a waitlist control [45]. A scoping review of meta-analyses revealed that CBM-A and CBM-I each have at least small effects in the expected directions on their proximal targets of attention and interpretation, respectively [46].

One randomized clinical trial (RCT) tested the effects of home-delivered, tablet-based FoR treatment in a patient population that serves as a model for applying the intervention to fearful cardiac patients [20]. This study used a well-matched sham control condition that allowed for participant blinding. Among cancer survivors, the intervention (combined CBM-A and CBM-I) reduced fear of cancer recurrence, as indexed by lastingly lower health worries 3 months post-intervention with a moderate between-group effect size (*g* = 0.54). As intended, CBMT also reduced interpretation biases, reducing the endorsement of threatening interpretations of ambiguous scenarios that were potentially related to cancer symptoms or prognosis (*g* = 1.65). Encouragingly, the faster patients rejected threatening interpretations of the ambiguous scenarios in the intervention group, the more steeply their health worries subsided, suggesting that the intervention’s effects on threat-related cognitions were likely responsible for the salutary changes in FoR. The study demonstrated that the intervention was feasible and acceptable. Although drop-out was 12%, over three quarters of patients who began the study completed the majority of training sessions, and 90% reported satisfaction with the intervention.

Because post-ACS patients could benefit from an intervention that reduces their heart-related fear by modifying negative cognitive biases, the present pilot study tested the feasibility of a CBMT intervention adapted for this particular patient population. Of note, this non-invasive, non-pharmacologic approach may even be preferred over counseling-based approaches for reducing distress in cardiac patients. As described in full detail in our published rationale-and-design paper, we adapted the stimuli and procedures of the successful cancer-related FoR intervention, as well as the cancer FoR target measure, for the current cardiac-related medical context [20, 24].

### Aims

We tested the feasibility of administering tablet-based CBMT to patients returning home from the hospital after suspected ACS as well as the feasibility of conducting a pilot RCT comparing CBMT to a sham control. In particular, we assessed six metrics: (1) the proportion of eligible patients who ultimately enrolled, (2) the proportion of enrolled patients who dropped out, (3) patient compliance with the at-home intervention sessions, (4) patients’ acceptability ratings of the intervention’s pleasantness/unpleasantness, (5) patients’ usability ratings of the ease/difficulty of usage of the intervention tablet-based procedures as a whole, and (6) patient’s task engagement with the at-home sessions (i.e., objectively recorded accuracy rates and response times).

Note that, due to early termination of data collection due to the COVID-19 pandemic, this preliminary study did not have an adequate sample size powered to address aims related to preliminary efficacy (e.g., reduction in FoR). Those aims are described in the design-and-rationale paper, and the findings are reported on ClinicalTrials.gov (ID: NCT03853213). Therefore, the present paper focuses exclusively on the feasibility of administering a tablet-based version of the intervention in the patient population and of conducting an RCT. We included planned feasibility metrics [24], but we did not pre-specify metrics for determining feasibility on ClinicalTrials.gov as this study was intended to be a preliminary efficacy trial.

## Methods

We provide a brief overview of the methods below for this double-blind randomized controlled trial with a 1:1 allocation ratio. For the full methodological details, see the published design-and-rationale paper for this study [24]. The study was approved by the Institutional Review Board of Columbia University Irving Medical Center (IRB-AAAR9458). All enrolled patients gave informed consent.

### Patient recruitment and eligibility

We recruited English- and/or Spanish-speaking patients who were admitted to the emergency department of Columbia University Irving Medical Center with a suspected diagnosis of ACS. Eligible patients reported elevated threat perceptions in the hospital (≥8 on the Emergency Department Threat Perceptions Scale) [47] and were prescribed at least one cardiovascular medication (i.e., antiplatelet, antihypertensive, or statin).

### Procedure

Enrolled patients were randomly assigned either to a CBMT intervention group or a sham control group (eight 30-min sessions over 4 weeks for both groups). Thus, the total time requested to devote to the tablet tasks was approximately four hours (~30 min/session) across the four weeks. Randomization was performed via SAS software (Version 9.4) independently by a data team member, and allocation to condition was done by the same data team member by loading the relevant tablet tasks (i.e., English or Spanish version of intervention or control) onto each patient’s study tablet. For details about randomization procedures, see the design paper [24]. All sessions were completed at home using an electronic tablet loaned by the study (Microsoft Surface Pro 6). Patients were provided with an electronic pill bottle to assess their medication adherence over 8 weeks. A baseline session occurred either in person in hospital prior to ACS discharge or at patients’ homes within 6 weeks of the ED visit. All patients completed a hands-on demonstration of the electronic tablet tasks and practiced opening a demonstration eCAP (electronic cap) medication bottle. Patients completed the baseline questionnaires at this session. The post-training session was conducted by phone or in person. Eight weeks after the baseline session, patients returned the medication adherence devices and exit questionnaires. All patients remained blinded to their assigned condition throughout their study participation, and all study staff remained blinded until data collection was completed for the final enrolled patient. Patients received monetary compensation at the time of study completion. Monetary completion for the tablet sessions was made dependent upon returning the tablet, and the compensation plan was tiered so that completing more sessions resulted in increasingly higher compensation per session.

### Intervention

Since our CBMT intervention had to be relevant for ACS patients’ FoR, we adjusted the language to be more directly relevant to ACS patients’ distinct fears. We developed language for cardiac-related threats in the CBMT-A task (e.g., “Mortality,” “Ambulance,” “Fatal,” “Stroke”), as well as ACS-relevant language for the CBMT-I task with ambiguous phrases that patients could perceive as either benign or threatening, depending on their interpretation bias (e.g., “You feel short-winded when walking up a flight of stairs,” “You feel a pain in your back for a few hours”). The details regarding the step-by-step adaptation of the CBMT paradigm for a fearful cardiac population, including the development of appropriate linguistic stimuli, are described in Birk at el. [24].

The intervention consisted of two tasks administered on an electronic tablet; the first targeting attention and the second targeting interpretation. Patients were asked to complete the tasks twice each week over the course of one month (for a total of 8 sessions or 16 tasks altogether). For those randomized to the intervention group, the CBMT-A task was designed to shift the spatial locus of attention toward neutral stimuli (e.g., “Holder”) and away from ACS threat-related stimuli (e.g., “Danger”). Patients were told to respond accurately with a rapid response rate by tapping the right or left side of the screen based on the letter they saw that replaced the word or phrase (“E” or “F”). In 95% of the trials in the intervention condition, the letter was presented on the portion of the screen in which the neutral stimulus had just appeared. Patients saw positive reinforcement (“Correct!!”) on the screen when they responded correctly and negative feedback (“Incorrect”) when they responded incorrectly (e.g., indicating that the letter presented was F when in fact it was E). The goal of the CBMT-I task was for patients to interpret information as non-threatening when presented with short scenarios which had ambiguous potential to be related or unrelated to ACS-related threats. In 90% of the trials, patients in the intervention group received positive reinforcement (“You are correct!”) when they interpreted prompts as non-threatening rather than threatening, and received negative feedback (Incorrect) when they interpreted the non-threatening prompts as threatening.

### Control

The two tasks assigned to patients in the sham control group were of a similar nature. However, sham CBMT for attention was designed *not* to increase patients’ attention towards either type of stimuli (neutral or ACS threat-related) as there was no systematic contingency between the location of the threat-related or neutral stimuli and the subsequent location of the target stimulus (i.e., the letter E or F). In other words, unbeknownst to patients, the program had assigned threat-related stimuli to appear in the same location as the subsequent target stimulus on 50% of trials and in a different location as the subsequent target stimulus on the other 50% of trials. Similarly, sham CBMT for interpretation was designed *not* to influence patients to interpret the ambiguous scenarios differently than they normally would. That was achieved by programming the stimuli such that there was no systematic contingency between the reinforcing feedback and the threat-related or benign interpretations that could be endorsed by patients. In other words, the program had assigned threat-related interpretations as the “correct” response on 50% of trials and benign interpretations as the “correct” response on the other 50% of trials.

### Feasibility outcomes and measures

The proportion of eligible patients who ultimately enrolled was assessed by simply dividing the number of approached patients who enrolled in the study (numerator) by the total number of eligible patients (denominator). We evaluate this proportion to reflect patients’ willingness to be randomized to a condition in this study of the tablet-based CBMT intervention. The proportion of enrolled patients who dropped out was similarly computed by dividing the number of patients who did not complete the study (numerator) by the total number of enrolled patients (denominator). Patient compliance with the at-home intervention was assessed by determining how many tablet tasks were completed in comparison to the total number of assigned tablet tasks, which was 8 at-home sessions.

Intervention acceptability and usability were assessed at the end of the study via an exit interview using 5-point Likert scales. For a measure related to *acceptability*, patients were asked, “In general, how would you describe the feelings you experienced while completing the tablet tasks?” and were presented with a 5-point response scale with displayed anchors of (1) “very unpleasant,” (3) “neither pleasant nor unpleasant,” and (5) “very pleasant.” For *usability*, patients were asked, “How easy or difficult was it for you to understand the instructions for the tablet tasks?” and were presented with a 5-point response scale with displayed anchors of (1) “very difficult,” (3) “neither difficult nor easy,” and (5) “very easy.” Additionally, at the end, patients were provided with an opportunity to comment on any aspect of their experience with the study in an open-ended question.

Task engagement was measured in the CBMT-A task through a combination of accuracy rate and response time. Accuracy rate is an indicator of whether or not the patient was actually engaging with and comprehending the material, as patients ultimately needed to identify which letter they saw on the screen (an E or an F), which should result in few, if any, inaccurate responses. Similarly, response time is an indicator of whether the patient was engaging and/or comprehending with the material because it is possible for a patient to respond and “complete” the task without reading. We compared response times to a minimum response time needed for a person to comprehend brief linguistic information, as well as their individual average response time as a barometer for unusually slow or fast responses.

### Other measures

We assessed other measures pertaining to study aims related to either preliminary efficacy of the intervention’s effects on putative mechanisms of behavior change or its effects on health behaviors. Note that these measures are not reported in the present paper because they do not pertain to feasibility of the CBMT intervention. (For findings related to these outcomes that are outside the present scope of feasibility testing, see NCT03853213 on ClinicalTrials.gov.) Specifically, we measured *FoR* in patients via a 19-item scale called Concerns about Recurrent ACS, which we adapted from a similar measure used for patients with breast cancer [48], patients’ *perceptions of time* via the Future Time Perspective scale [49], and their *sensitivity to contextual clues* via the Context Sensitivity Index [50]. We assessed *medication adherence* objectively via pill bottle openings (Information Mediary Corp., Ottawa, Canada) and via assessing the self-reported extent of non-adherence [51, 52] and *physical activity* via the International Physical Activity Questionnaire [53]. We also collected basic demographic information, baseline clinical characteristics, baseline PTSD symptoms [PTSD Checklist-Civilian; [54]], baseline PTSD symptoms due to a non-ACS trauma [PTSD Checklist-Specific; [55]] and depressive symptoms [8-item Patient Health Questionnaire; [56]].

### Sample size

Although a power analysis was conducted for the overall RCT objectives as reported in the design paper [24], no power analysis was performed to determine the needed sample size for the feasibility objectives. Instead, we determined that evaluating the percentage of patients who enrolled would yield a meaningful estimate of our main feasibility metric given a denominator of 49 patients screening eligible from the parent study. Our secondary feasibility metric of the percentage of patients who dropped out is necessarily dependent on the number enrolled, but we deemed that reporting the percentage out of the denominator of 26 enrolled may provide an informative preliminary estimate for future researchers. We determined that the feasibility measure of percentage tablet return could yield a meaningful estimate with the denominator of 20 who were given tablets. The other quantitative feasibility outcomes—tablet task compliance and exit interview ratings—all involved descriptive statistics for which we deemed the sample of 20 analyzed participants sufficient to provide useful information to understand the feasibility of the intervention in this patient population.

### Statistical analysis

All feasibility objectives were assessed using descriptive statistics that depended on the type of data. Specifically, counts and percentages are presented below for eligible patients who enrolled, enrolled patients who dropped out, and patients completing at least one requested at-home session. Means (*M*) and standard deviations (*SD*) are presented for acceptability/pleasantness ratings, usability ratings, accurate tablet task trials, and valid tablet task trials in terms of response time. Each of these metrics will be considered in reference to the relevant comparison point described below in the Progression Criteria section.

### Progression criteria

Progression criteria that would indicate that continuing with a larger clinical trial would be feasible are as follows (each point corresponding to an aim in the “Aims” section of the Introduction): (1) proportion of eligible patients who enrolled of at least 25%, which is an amount comparable to previous CBMT research in a population of medical patients [20], (2) a drop-out rate of no more than 12% [20], (3) at least 50% of patients complying with the intervention by completing at least one of the requested at-home sessions, (4) average acceptability/pleasantness ratings of at least 4 out of 5 points, (5) average usability ratings of at least 4 out of 5 points, and (6) average across patients of at least 90% accurate trials and average of at least 90% trials having response times that were greater than 150 ms but within 4 standard deviations above or below the patient’s mean response time.

## Results

Twenty-six patients enrolled in the study. The mean age was 59.15 years (*SD* = 14.57; range: 25–93; 10 [38.5%] patients aged ≥ 65). Patients were diverse (20.0% Black, 40.0% Hispanic, 66.0% completed less than college graduation). Patients reported mean threat perceptions (possible range: 0–6) of 2.31 points (*SD* = 0.73). Table 1 presents patient characteristics at baseline by group.

**Table 1:** Patient characteristics at baseline

**Characteristic**	**Intervention (*n* = 11)**	**Control (*n* = 9)**	**Total (*n* = 20)**
**Age (mean, *SD*, in years)**	62.27 (16.67)	55.33 (11.27)	59.15 (14.57)
**Female sex**	6 (54.55%)	4 (44.44%)	10 (50.00%)
**Race/ethnicity**			
Black	3 (27.27%)	1 (11.11%)	4 (20.00%)
White	2 (18.18%)	3 (33.33%)	5 (25.00%)
Other	0 (0.00%)	1 (11.11%)	1 (5.00%)
Dominican	1 (9.09%)	1 (11.11%)	1 (5.00%)
Hispanic or Latino	5 (45.45%)	3 (33.33%)	8 (40.00%)
**Education**			
Some high school	3 (33.33%)	2 (22.22%)	5 (25.00%)
High school diploma/GED	2 (22.22%)	2 (22.22%)	4 (20.00%)
Some college	2 (22.22%)	2 (22.22%)	4 (20.00%)
College graduate	2 (22.22%)	1 (11.11%)	3 (15.00%)
Graduate school/professional school	2 (22.22%)	2 (22.22%)	4 (20.00%)
**Charlson comorbidity index (mean, *SD*)**	1.45 (1.51)	2.44 (2.40)	1.90 (1.97)
**PTSD symptoms (PCL-5) (mean, *SD*)**	18.23 (13.51)	22.83 (20.67)	20.36 (16.58)
**Depressive symptoms (PHQ-8) (mean, *SD*)**	8.94 (5.81)	6.75 (3.45)	7.97 (4.91)

Data are presented for all patients who were given study tablets corresponding to the intervention or control condition, regardless of how many tablet tasks they subsequently completed. PTSD symptoms and depressive symptoms were assessed at baseline during the hospitalization period that occurred within days of the first study visit. PTSD symptoms were keyed to the suspected cardiac event and the surrounding hospitalization

*PCL-5* PTSD Checklist for DSM-V, *PHQ-8* 8-item Patient Hospitalization Questionnaire, *PTSD* Posttraumatic stress disorder

**Fig. 1:**
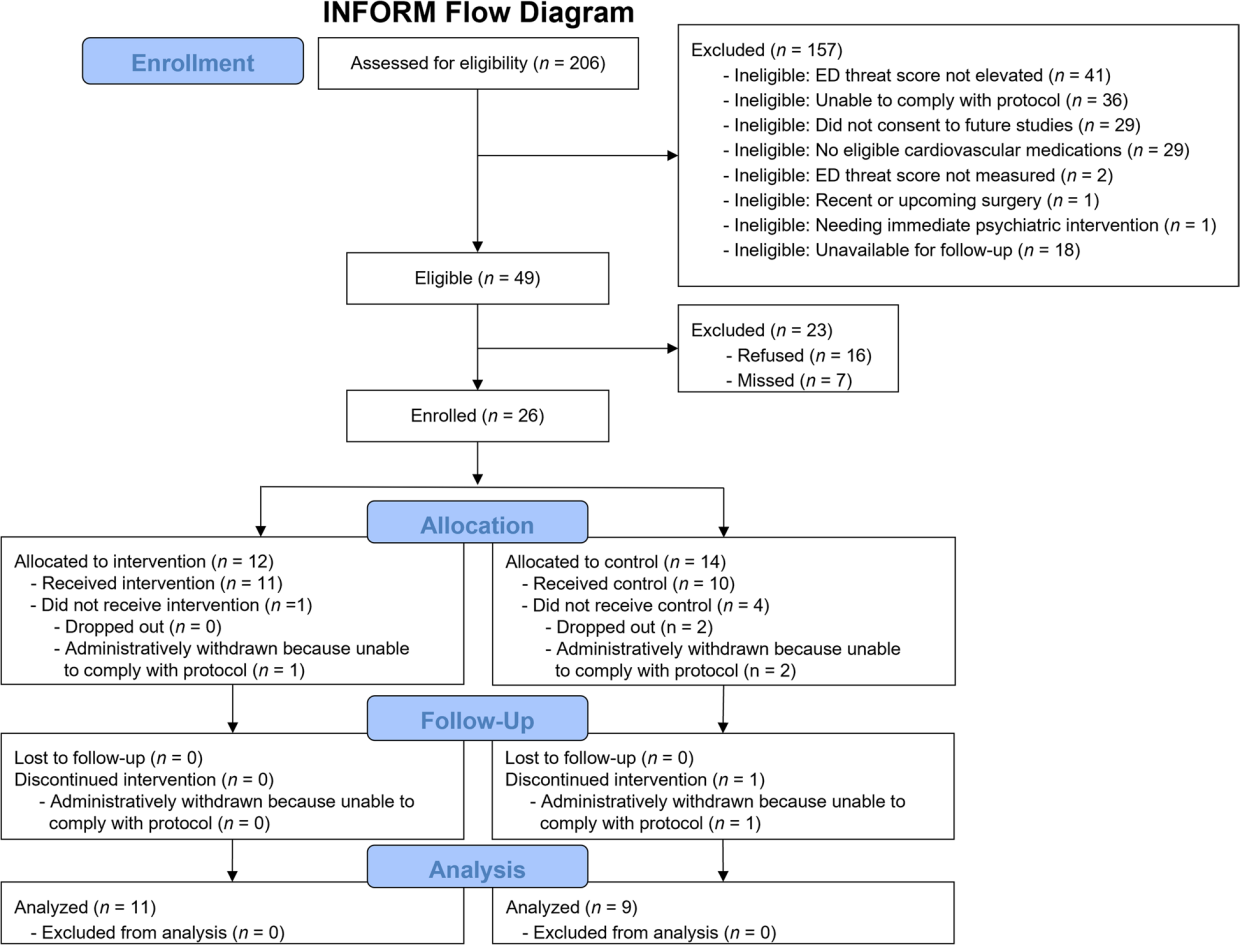
Patient flow diagram. Six of the enrolled patients did not have data and thus were not analyzed for acceptability analyses. ED threat refers to the Emergency Department Threat Perceptions Scale

Regarding the first feasibility metric, slightly over one half of all patients who had been screened eligible via data in the parent study enrolled in the study (53.1%; 26/49). Regarding the second feasibility metric, only 7.7% of the randomized patients (2/26) terminated their participation before completion of the study. Patients’ reasons for attrition were imminent plans to relocate outside the USA and a desire to focus on their health/recovery. In both cases, the patients discontinued participation after the informed consent process, but before the completion of the initial study session. In addition, 15.4% of enrolled patients (4/26) were administratively withdrawn from the study for not being able to comply with its procedures. Despite extensive guidance from the study team, they were unable to complete the tasks due to difficulties using the tablet and/or an inability to process linguistic information in a timely manner. Of the 206 patients from the parent study we screened for eligibility, 41 were ineligible due to low ED threat perception scores (i.e., lack of evidence of elevated baseline fear), 36 were ineligible due to being unable to comply with the protocol (either self-selected or indicated during screening based on conditions such as visual impairment), 29 had indicated during the parent study that they were not interested in being approached for future research studies, 29 were not prescribed any eligible cardiovascular medications, and 18 were unavailable for follow-up. Figure 1 depicts the CONSORT flow diagram for the intervention and control groups.

### Tablet return and compliance with tablet tasks

**Fig. 2:**
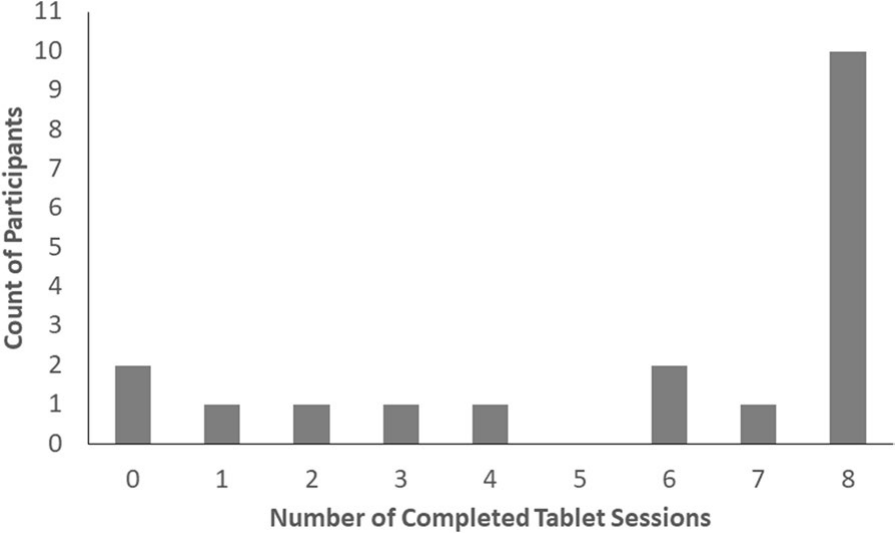
Compliance with assigned tablet tasks among the 19 patients who returned tablets. A session was considered complete if all trials in the CBM-A and CBM-I tasks were completed within one session of either the intervention or control condition. As shown above, most patients who returned tablets completed all assigned tablet tasks

Nineteen of the 20 patients returned their tablets. Although each patient was initially given a single tablet, two patients were given a replacement tablet because of technical difficulties, and these two patients returned both of their tablets. As one tablet was not returned due to circumstances related to the COVID-19 pandemic, we were able to determine compliance with the tablet tasks for 19 patients. Several patients made special trips to the medical center to hand-deliver the tablet even though they could have returned it for free by mail using prepaid study packaging. Among the 19 patients who did return tablets, 89.5% (17/19) patients completed one or more of the assigned at-home sessions, 68.4% (13/19) of patients completed the majority (five or more) of the assigned at-home sessions, 52.6% (10/19) achieved perfect compliance (i.e., 8 or more at-home sessions), and 10.5% (2 patients) did not complete any sessions even though they returned the tablets. Figure 2 shows patient compliance for the at-home intervention procedures.

 Regarding patient task engagement, the mean (*SD*) percentage of accurate CBMT-A trials across groups was generally high but with substantial variability: 91.8% accuracy (*SD* = 13.6%; range: 43.8–100.0%). Notably, given that 50% represents chance-level accuracy, two patients (both in the intervention group) showed evidence of possible disengagement: 43.8% and 77.3% accuracy respectively. All other patients responded accurately on more than 90% of trials. Invalid response times on CBMT-A trials were defined as either being (1) <150 ms (i.e., too fast to reflect a behavioral response to the perceived stimuli) or (2) not within 4 standard deviations above or below the patient’s mean response time. On the basis of these invalid CBMT-A trials, the mean (*SD*) percentage of valid trials across patients and across groups was acceptably high: 94.9% valid (*SD* = 7.1%; range: 78.1–100.0%).

### Exit interview ratings and feedback

Across groups, patients rated their experience using the tablet-based intervention as generally pleasant (*M* = 4.20, *SD* = 0.92; range: 3 [*Neither pleasant nor unpleasant*] to 5 [*Very pleasant*]). Patients in the intervention (*M* = 4.17, *SD* = 0.98; range: 3–5) and control (*M* = 4.25, *SD* = 0.96; range: 3–5) groups provided similarly high ratings of pleasantness. Across groups, patients rated the intervention procedures as a whole (i.e., the tablets and the assigned tasks) as easy to use (*M* = 4.60, *SD* = 0.70; range: 3 [*Neither difficult nor easy*] to 5 [*Very easy*]). Patients in the intervention (*M* = 4.33, *SD* = 0.82; range: 3–5) and control (*M* = 5.00, *SD* = 0.00; range: 5–5) groups rated the intervention procedures as easy or very easy.

In free-text feedback responses, patients in the intervention group reported the following information. One patient reported that the content of the tablet tasks was confusing, a second patient reported that it was not always clear whether the device was working properly or not, a third patient reported that it was sometimes hard to understand the questions and answers, and a fourth patient reported that the tablet froze a few times. Patients in the control group reported the following information. One patient thanked the research team, and a second patient recommended that sign language be somehow incorporated for deaf people in the tablet tasks and reported being impatient with the tablet not starting properly sometimes.

## Discussion

The tablet-based CBMT intervention was moderately acceptable to eligible patients with suspected ACS. Specifically, under a third of eligible patients declined to participate in the study. Furthermore, over a third of eligible patients and nearly two thirds of enrolled patients ultimately chose to engage with the at-home tablet tasks by completing at least one such task. Encouragingly, of those who completed *any* of the at-home tasks, most patients completed *all* of the assigned tasks. For the most part, patients completed the intervention and control tasks as requested. Two thirds of patients who remained active in the study (i.e., did not withdraw and were not administratively withdrawn by study staff) completed the majority of tablet tasks. Furthermore, despite the high level of sustained attention required and the presence of potentially threatening cardiac-related information in the tasks, patients rated the intervention overall to be pleasant and easy to use. These findings are notable given that the total time required to complete the eight requested sessions was considerable.

Our feasibility indices compare favorably with other research using a CBMT intervention. Notably, 53% of eligible patients enrolled in our study whereas only 26% of eligible patients enrolled in the parallel intervention in patients with breast cancer [20], a result which supports that our patient population found the premise of the intervention reasonably acceptable. However, a similar percentage of patients in both studies completed any portion of the intervention: 65% (17/26) of enrolled patients in the present study and 71% in the previous study with breast cancer patients. The overall drop-out rate across groups (7.6%, 2/26) was slightly lower than that for the parallel intervention in patients with breast cancer (12% drop-out) [20] and lower than that for a smartphone based CBM-A intervention for social anxiety (33%) [57]. Our complete compliance rate of 53% for the tablet tasks was somewhat lower than the 66% complete compliance rate observed in a CBMT trial that used a touchscreen version of CBMT with a mobile app for a different population recruited from a hospital (women receiving prenatal treatment who were interested in anxiety and stress reduction) [33].

The intervention had many strengths. The adapted CBMT intervention and study procedures were designed to be as inclusive and accessible as possible. First, we used a consecutive sample of patients with suspected ACS to lower the risk of selection bias. Second, patients were not require to have prior experience with health applications or to have internet access. Third, the intervention was completed at home at convenient times that were chosen by patients. Fourth, confusion about how to start the tablet tasks was nearly eliminated because the tablets were programmed to launch the task automatically upon being turned on. Fifth, the hands-on demonstration of tablet tasks in the first study session ensured that patients understood the tablet task procedures. Sixth, all study materials were professionally translated into Spanish to increase accessibility. Seventh, when needed and possible, the study team enrolled and occasionally conducted study visits in patients’ homes, although this particular strength may not be scalable for a larger efficacy trial or in clinical practice. Finally, the risk of bias due to blinding of patients and study personnel was low because of the well-matched nature of the intervention tasks that consisted of identical stimuli that only differed in terms of the programmed contingencies and feedback (see design paper) [24].

The high rate of device return was overall a sign of feasibility. This aspect of feasibility must be understood in light of monetary compensation being linked to completion of tablet tasks and return of the tablets. Thus, compensation may have played a role in both the high tablet task completion rate (see Figure 2) and the high tablet return rate. Feasibility of device return in a larger clinical trial could be hampered by difficulties scaling up the return procedures, especially for samples with a wider geographic distribution.

Despite many strengths, the study also encountered substantial hurdles. Most notably, the study only reached about 25% of its anticipated *N*, thereby rendering results of the main preliminary efficacy aims insufficiently powered to test properly. Thus, the study should be considered as a pilot and feasibility study of a randomized controlled trial. Indeed, it was the first known tablet-based CBMT intervention of its kind applied to this specific medical population. Upon first glance, an enrollment of 26 patients out of 49 eligible patients appears somewhat low. Notably, we received only 16 total refusals, which leads us to believe that the study was largely acceptable in light of the relatively high ratio of consenting patients to refusing patients: 1.63 (i.e., 26 consenting/16 refusing). Another challenge to recruitment was the COVID-19 pandemic, which necessitated the halt of recruitment for this and other studies at the medical center in March 2020. One notable consequence of this early termination is that the 19-item Concerns About Recurrent ACS Scale remains unvalidated, which points to an important direction for future research. In addition, patients who had difficulties with the technology or were unable to process the visually presented linguistic information at a sufficient speed were either not enrolled or administratively withdrawn, which may impact the generalizability of this tablet-based CBMT intervention for this patient population. Finally, despite the demonstrated association between the transdiagnostic construct of FoR and anxiety [23], the present study did not have a measure of anxiety, although it did assess other measures of psychological distress, including PTSD and depressive symptoms. Future research should test the extent to which interventions that successfully reduce FoR may also improve multiple indices of psychological distress.

## Conclusion

The tablet-based CBMT intervention showed reasonable feasibility and acceptability given that two thirds of the study patients completed the at-home study tasks and responded accurately in more than 90% of the trials. Nevertheless, preliminary efficacy could not be assessed due to low recruitment and the COVID-19 pandemic ending the study prematurely. The authors recommend that CBMT remain an area of future investigation as a means of decreasing fear of disease progression in cardiac patients in both inpatient and outpatient settings. Given the burden on patients of potentially needing to learn a new technology and the logistical burden on research staff of having to retrieve tablets, the authors recommend pursuing CBMT with HTML-based technology where patients use their own devices (e.g., smartphone, laptop), perhaps via E-Prime Go [58] or a similar HTML-based stimulus presentation service in a larger clinical trial.

## Data Availability

The datasets analyzed in the current study are available from the corresponding author on reasonable request.
